# LINC01806 mediated by STAT1 promotes cell proliferation, migration, invasion, and stemness in non-small cell lung cancer through Notch signaling by miR-4428/NOTCH2 axis

**DOI:** 10.1186/s12935-022-02560-8

**Published:** 2022-05-22

**Authors:** Shangxiao Huang, Shixiong Liang, Jianfeng Huang, Penghui Luo, Dunchang Mo, Hanlei Wang

**Affiliations:** 1 Department of Radiotherapy, The Third Affiliated Hospital of Guangxi Medical University, No.13 Dancun Road, Nanning, 530031 Guangxi China; 2 Department of Radiotherapy, Cancer Hospital Affiliated to Guangxi Medical University, Nanning, 530021 Guangxi China

**Keywords:** Non-small cell lung cancer, STAT1, LINC01806, miR-4428, NOTCH2

## Abstract

**Background:**

Non-small cell lung cancer (NSCLC), the most primary lung cancer subtype, threatens human health globally. Long non-coding RNAs (lncRNAs) have been uncovered to affect multiple cancers progression. Nevertheless, the specific function of long intergenic non-protein coding RNA 1806 (LINC01806) in NSCLC remains elusive.

**Methods:**

RT-qPCR and western blot were involved in this study. The influence of LINC01806 on NSCLC was assessed by in vitro and in vivo assays. Via ChIP, RNA pull down, RIP, and luciferase reporter assays, the in-depth cellular mechanisms of LINC01806 in NSCLC were explored.

**Results:**

LINC01806 expression was high in NSCLC cell lines. Functionally, LINC01806 knockdown impeded cell proliferation, migration, invasion, and stemness, along with tumor growth. As for its mechanism, signal transducer and activator of transcription 1 (STAT1) activated LINC01806 transcription in NSCLC. Furthermore, LINC01806 sequestered microRNA-4428 (miR-4428) to enhance notch receptor 2 (NOTCH2) expression, thus activating Notch signaling pathway. Finally, in vitro and in vivo assays jointly validated that LINC01806 exerted its function in NSCLC development via miR-4428/NOTCH2 pathway.

**Conclusion:**

LINC01806 enhanced NOTCH2 expression to stimulate Notch signaling via sponging miR-4428, thereby facilitating NSCLC progression, which provided a novel mechanism for NSCLC therapeutic approaches.

**Graphical Abstract:**

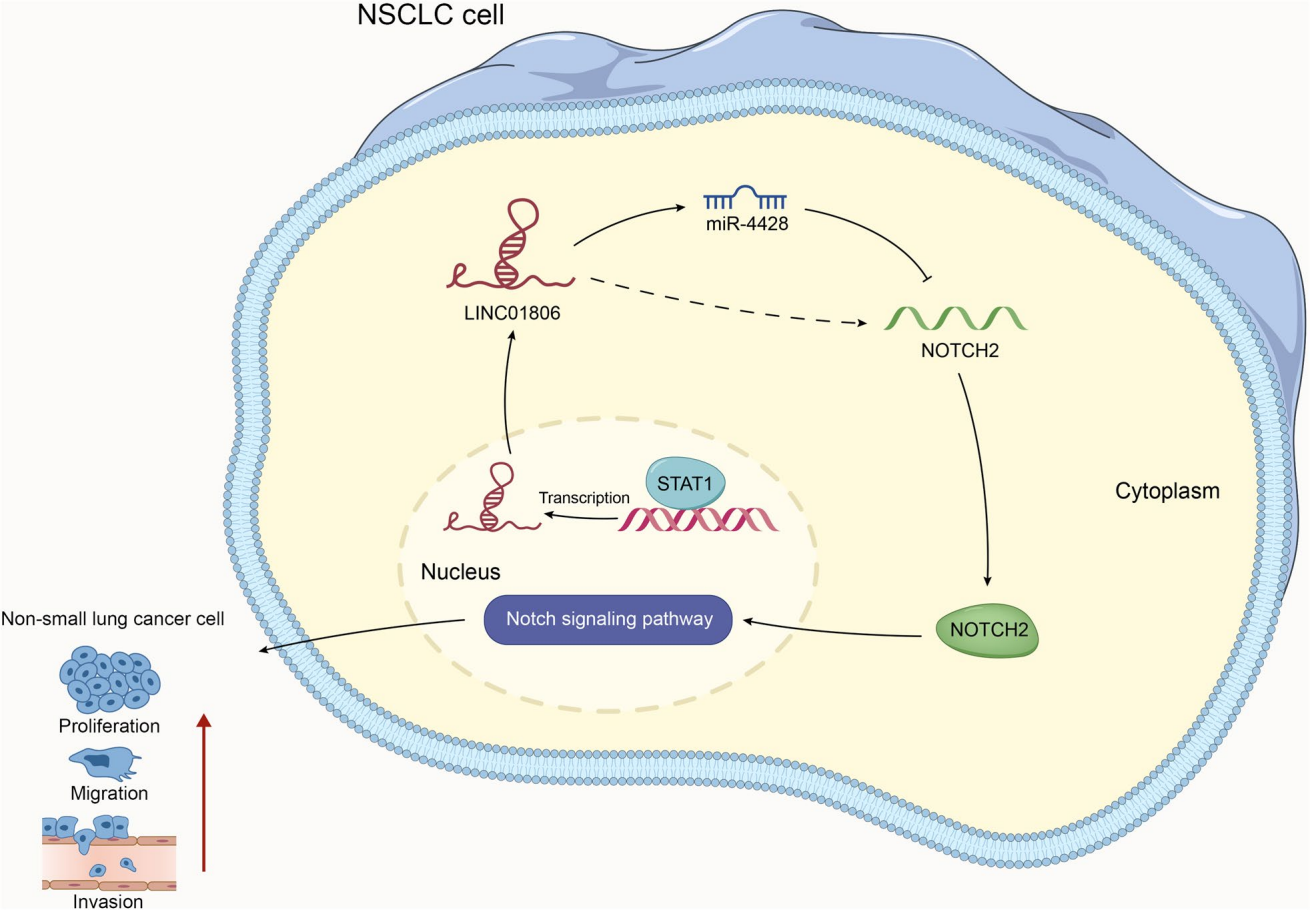

**Supplementary Information:**

The online version contains supplementary material available at 10.1186/s12935-022-02560-8.

## Background

As the dominant subtype of lung cancer, non-small cell lung cancer (NSCLC) has been identified as a deadly disease for a long time [1, 2]. In recent years, advancements in NSCLC treatment have been achieved. Nevertheless, the median survival for NSCLC patients is still poor [3]. Hence, seeking for novel biomarkers which are linked with the development and progression of NSCLC is especially vital [4].

Long non-coding RNAs (lncRNAs) represent a class of transcripts whose lengths are more than 200 nucleotides. Emerging research has suggested that lncRNAs participate in cancer progression and metastasis [5, 6]. For example, Du et al. have pointed out LINC00319 leads to the development of ovarian cancer via the modulation of miR-423-5p/NACC1 axis [7]. Meng et al. have discovered the inhibitory role of SNHG6 in colorectal cancer metastasis [8]. As reported by Yang et al., PVT1 has the promoting influence on prostate cancer tumor growth [9]. Moreover, many reports have also demonstrated the correlation between lncRNAs and NSCLC development [10]. For example, another research work published by Jia et al. have suggested that MAFG-AS1 acts as an oncogene in NSCLC through binding with miR-339-5p and enhancing MMP15 expression [11]. Cui et al. have proposed that SNHG1 prompts the progression of NSCLC through activating Wnt/β-catenin signaling pathway [12]. In addition, Xie et al. have demonstrated that LINC01234 mediated by SP1 functions as a ceRNA to facilitate NSCLC progression [13]. However, as a novel lncRNA, LINC01806 is scarcely investigated in diseases and remains to be deeply studied.

The current study targeted at unveiling the biological impact of LINC01806 (2,348 bp) and exploring the deep-going mechanism of LINC01806 in NSCLC.

## Methods

### Cell culture

NSCLC cell lines (A549, HCC827, H1650 and H1299) along with human normal lung epithelial cell line (BEAS-2B) were all obtained from American Type Culture Collection (ATCC; Manassas, VA, USA). Among them, HCC827, H1650 and H1299 cell lines were maintained in RPMI-1640 medium (61870127, Gibco, Gaithersburg, MD, USA). A549 cell line grew in F-12 K medium (21127022, Gibco), whereas BEAS-2B cell line was cultivated in bronchial epithelial cell growth medium (BEGM; CC-3170, LONZA). Media were mixed with 10% FBS (16140071, Thermo Fisher Scientific, IL, USA). Incubators were maintained at 37 °C with 5% CO_2_.

### Cell transfection

The shRNAs for knocking down LINC01806 or STAT1 along with non-target negative control (sh-NC) were designed with the aid of GenePharma (Shanghai, China). Moreover, pcDNA3.1 vectors (GenePharma) covering the cDNA sequences of STAT1 or NOTCH2 were used for gene overexpression. MiR-4428 mimics and the corresponding NC mimics were produced by GenePharma. Moreover, micrOFF™ miRNA inhibitor for miR-4428 inhibition, along with its NC inhibitor was also procured from RiboBio. The transfection of indicated plasmids was completed using Lipofectamine 2000 (11668019, Invitrogen, Carlsbad, CA, USA). Sequences of shRNAs used in this study were provided in Additional file 3: Table S1.

### Quantitative reverse transcription PCR (RT-qPCR) analysis

With the use of TRIzol Reagent (15596026, Invitrogen), total RNA was extracted. Next, the cDNA of RNAs were obtained through reverse transcription utilizing M-MLV reverse transcriptase (M1701, Promega, Madison, WI, USA). For qPCR detection, GoTaq qPCR Master Mix (A6001, Promega) was applied. The expression levels of involved genes were calculated via 2^−ΔΔCt^ method. GAPDH or U6 functioned as the internal reference. Analysis was independently verified for three times. Primer sequences were displayed in Additional file 1: Table S2.

### Colony formation assay

In this assay, NSCLC cells (500 cells per well) were plated into 6-well plates. Following 14-day incubation, cells underwent fixation by ethanol and staining by 0.1% crystal violet. The quantity of obviously noticed colonies (> 50 cells) was calculated manually. The experiment was independently conducted for three times.

### 5-ethynyl-20-deoxyuridine (EdU) staining assay

Cells were plated in 96-well plates with 5 × 10^4^ cells per well. Then, cells were incubated with EdU solution (C10310, Ribobio). Nuclear part of cells was stained by DAPI (D9542, Sigma-Aldrich). After that, images were captured under the fluorescence microscope (Olympus, Tokyo, Japan). The independent experiment was performed in triplicate.

### Transwell assay

Cells were plated into the top compartment of transwell insert, and the chambers used in invasion assay were specially covered with Matrigel (356234, BD Biosciences San Diego, CA, USA). As to transwell migration assay, Matrigel-free chambers were utilized. Then the lower compartment contained culture medium mixed with 10% FBS. After 24 h, cells first fixed by 4% formaldehyde were stained by crystal violet (ab232855, Abcam) for calculation. The assay was independently performed for three times.

### Sphere formation assay

NSCLC cells (1000 cells/well) were cultured in 24-well ultra-low attachment plates (Corning, Shang, China) with the use of MammoCult™ Human Medium Kit (Cat#05620, Stemcell Technologies, Vancouver, BC, Canada). Following 10-day incubation, the quantity and size of spheroids were evaluated under a microscope. The sphere formation efficiency of experimental groups was determined relative to corresponding control groups, and cell spheres with diameter larger than 75 μm were counted. The assay was independently performed for three times.

### Flow cytometry analysis

For the assessment of cell cycle, transfected NSCLC cells were fixed by 75% ethanol at 4 °C for 4 h. Afterwards, PBS was applied to rinse the cells, and PI was employed to stain cells. Following the 30-min cultivation at 4 °C, cells were subject to flow cytometer analysis for detecting cell cycle.

### Subcellular fractionation

Cytoplasmic and Nuclear RNA Purification Kit (NORGEN, Thorold, ON, Canada) was utilized as per user guide. RT-qPCR was exploited to quantify LINC01806 level in nuclear and cytoplasmic fractions. GAPDH or U6 respectively served as the internal reference for cytoplasm or the nucleus. The assay was independently performed for three times.

### Fluorescent in situ hybridization (FISH)

Following 15-min fixation with 4% paraformaldehyde (PFA) at 37 °C and permeabilization by 0.5% Triton X-100, NSCLC cells were incubated with LINC01806-specific FISH probes in hybridization buffer. Subsequently, NSCLC cells were counterstained with the use of Hoechst solution (62249, Thermo Fisher Scientific). Images were photographed under a confocal laser microscope (Olympus). The assay was independently performed three times.

### RNA pull down assay

Lysis buffer was utilized to lyse NSCLC cells. Next, obtained cell lysates were incubated with biotin-labeled LINC01806 probe. Afterwards, magnetic beads were put into the mixture. One night later, RNA precipitated by beads was purified for RT-qPCR detection. The assay was independently done in triplicate.

### RNA immunoprecipitation (RIP)

RNA-Binding Protein Immunoprecipitation Kit (Millipore, Burlington, MA, USA) was involved in this assay. Manufacturer’s instructions were strictly followed. Transfected NSCLC cells were lysed with RIP lysis buffer. Next, magnetic beads linked with anti-Ago2 (Abcam) or anti-IgG (Abcam), were added into cell lysates. At last, the precipitated RNA complexes were purified for RT-qPCR detection. The assay was conducted in triplicate.

### Chromatin immunoprecipitation (ChIP)

The EZ ChIP™ Chromatin Immunoprecipitation Kit (Millipore, Burlington, MA, USA) was involved in this assay. Cross-linked chromatin DNA underwent sonication to procure chromatin fragments, and then mixed with anti-STAT1 (Abcam) antibody and anti-IgG antibody (Abcam). The precipitated DNA fragment was analyzed by qPCR. The experiment was performed in triplicate.

### Luciferase reporter assay

The pGL3 vectors (E1751, Promega) covering wild type (Wt) or mutant type (Mut) LINC01806 promoter sequence were constructed. Next, the obtained constructs (pGL3-LINC01806 promoter-Wt/Mut) were co-transfected with pcDNA3.1/STAT1 or the control vector into NSCLC cells. The sequences of LINC01806/NOTCH2 3’-UTR-Wt/Mut were sub-cloned into pmirGLO dual-luciferase vectors (E1330, Promega) to generate pmirGLO-LINC01806 Wt/Mut and pmirGLO-NOTCH2 3’-UTR-Wt/Mut vectors, separately. Later, the recombinant plasmids were co-transfected with miR-4428 mimics or NC mimics into NSCLC cells. Forty-eight hours later, the luciferase activity was examined via Dual-Luciferase Reporter Gene Assay Kit (Beyotime, Shanghai, China). This assay was conducted in triplicate.

### Western blot analysis

Total protein was separated from the cell lysates with the use of SDS-PAGE, which was further transferred to PVDF membranes (Thermo Fisher Scientific). Subsequently, involved primary antibodies were added onto membranes for incubation overnight at 4 °C. After PBS rinsing for 3 times, secondary antibodies (1/2000) were added for 1-h incubation. The primary antibodies against Oct4 (1/1000), Nanog (1/1000), SOX2 (1/1000), NOTCH2 (1/2000), HES1 (1/1000), HES6 (1/1000), and β-actin (1/5000) were purchased from Abcam. Chemiluminescence system (GE Healthcare, Chicago, USA) was applied for protein detection. The assay was conducted for three times.

### In vivo experiments

After transfection with sh-NC, sh-LINC01806#1, sh-LINC01806#1 + NC antagomir, sh-LINC01806#1 + antagomir-4428, sh-LINC01806#1 + pcDNA3.1 or sh-LINC01806#1 + pcDNA3.1/NOTCH2, NSCLC cells (1 × 10^7^) were subcutaneously injected into nude mice (4–5 weeks of age). The volume of xenograft tumors was calculated every 5 days. After 30–day monitoring, the mice were anesthetized following intraperitoneal injection of pentobarbital sodium (50 mg/kg), and later sacrificed. Ultimately, the xenograft tumors were excised. The weight of excised tumors was also measured. Mice were purchased from Institute of Model Fauna, Nanjing University. All animal studies acquired the approval from the ethic committee of the Third Affiliated Hospital of Guangxi Medical University.

### Immumohistochemical (IHC) staining

Xenografts acquired from in vivo experiments were subjected to IHC assay. For this experiment, xenografts were treated by 4% PFA and then embedded in paraffin. Subsequently, tissues were sectioned into fragments with a thickness of around 5 μm. Next, the sections were subjected to incubation with anti-SOX2 (1/100), anti-Ki67 (1/500) or anti-Oct4 (1/250) antibody at 4 °C overnight. Later, the HRP-conjugated secondary antibodies (1/2000) were added. Peroxidase was visualized by DAB buffer (ACC500, ScyTek). After counterstaining the nuclei via hematoxylin, cells in the sections were captured under a microscope.

### In situ hybridization (ISH)

The xenograft tumor tissues obtained from the in vivo experiments were fixed with 4% PFA. After fixation, the tumor tissues were hybridized with digoxigenin (DIG)-labeled LINC01806 probe overnight at 4 °C. The signal of the probe was detected under the light microscopy.

### Statistical analysis

The gathered experimental data were processed via SPSS 22.0 statistical software and then displayed as mean ± standard deviation (SD). Analyses of differences between two or more groups were conducted via Student’s t-test or ANOVA (one-way or two-way). Each independent experiment was performed in triplicate. Data differences had statistical significance when P value was smaller than 0.05.

## Results

### LINC01806 depletion hinders proliferative, migratory and invasive capabilities of NSCLC cells

**Fig. 1:**
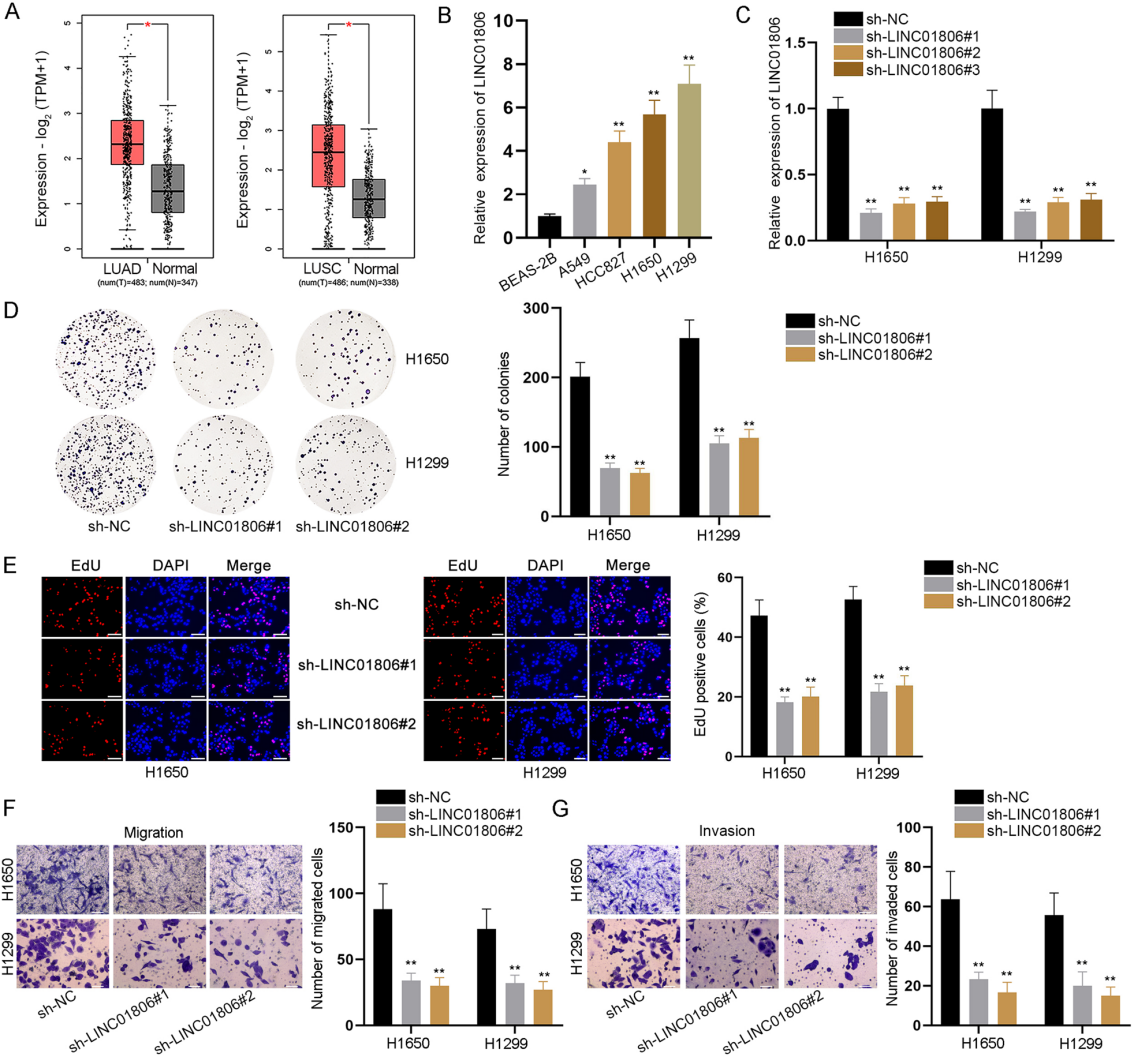
LINC01806 depletion hinders proliferation, migration and invasion of NSCLC cells. **A** GEPIA database demonstrated the level of LINC01806 in LUAD and LUSC tissues. **B** RT-qPCR analysis was applied to examine LINC01806 expression level in NSCLC cells and normal cells. **C** The knockdown efficacy of sh-LINC01806 was tested through RT-qPCR in H1650 and H1299 cells. **D**, **E** The proliferative ability of LINC01806-depleted NSCLC cells was detected in colony formation and EdU assays (scale bar = 100 μm) when LINC01806 was silenced or not. **F**, **G** Transwell assay (scale bar = 100 μm) was done to observe changes of NSCLC cell migration and invasion before and after LINC01806 deficiency. *P < 0.05, **P < 0.01

As demonstrated on GEPIA database (http://gepia.cancer-pku.cn/), LINC01806 expression level was extraordinarily high in lung adenocarcinoma (LUAD) and lung squamous cell carcinoma (LUSC) tissue samples in contrast to corresponding normal tissue samples (Fig. 1A). In view of that LUAD and LUSC are two main types of NSCLC [14], we inferred that LINC01806 might be an oncogene in NSCLC. Likewise, RT-qPCR analysis exhibited that compared with the normal BEAS-2B cells, LINC01806 was dramatically up-regulated in NSCLC cell lines (A549, HCC827, H1650, and H1299) (Fig. 1B). Since the expression of LINC01806 was higher in H1650 and H1299 cells compared to other NSCLC cell lines, we involved them in the subsequent experiments. Before loss-of-function assays, sh-LINC01806#1/2/3 vectors were adopted to cut down LINC01806 expression in H1650 and H1299 cells, and cells successfully transfected with sh-LINC01806#1/2 were applied subsequently (Fig. 1C). Colony formation and EdU assays were conducted, and the data suggested that cell proliferation was obstructed due to LINC01806 knockdown (Fig. 1D, E). In addition, cell migration and invasion were tested in transwell assays. The findings validated that silencing LINC01806 led to suppression on migration and invasion of NSCLC cells (Fig. 1F, G). Based on flow cytometry analysis, LINC01806 knockdown increased the proportion of cells at G0/G1 phase, while decreasing that of cells at S and G2/M phases (Additional file 1: Fig. S1A). In sum, all these data support that LINC01806 exacerbates NSCLC cell malignant behaviors.

### LINC01806 contributes to stemness of NSCLC and BEAS-2 cells

**Fig. 2:**
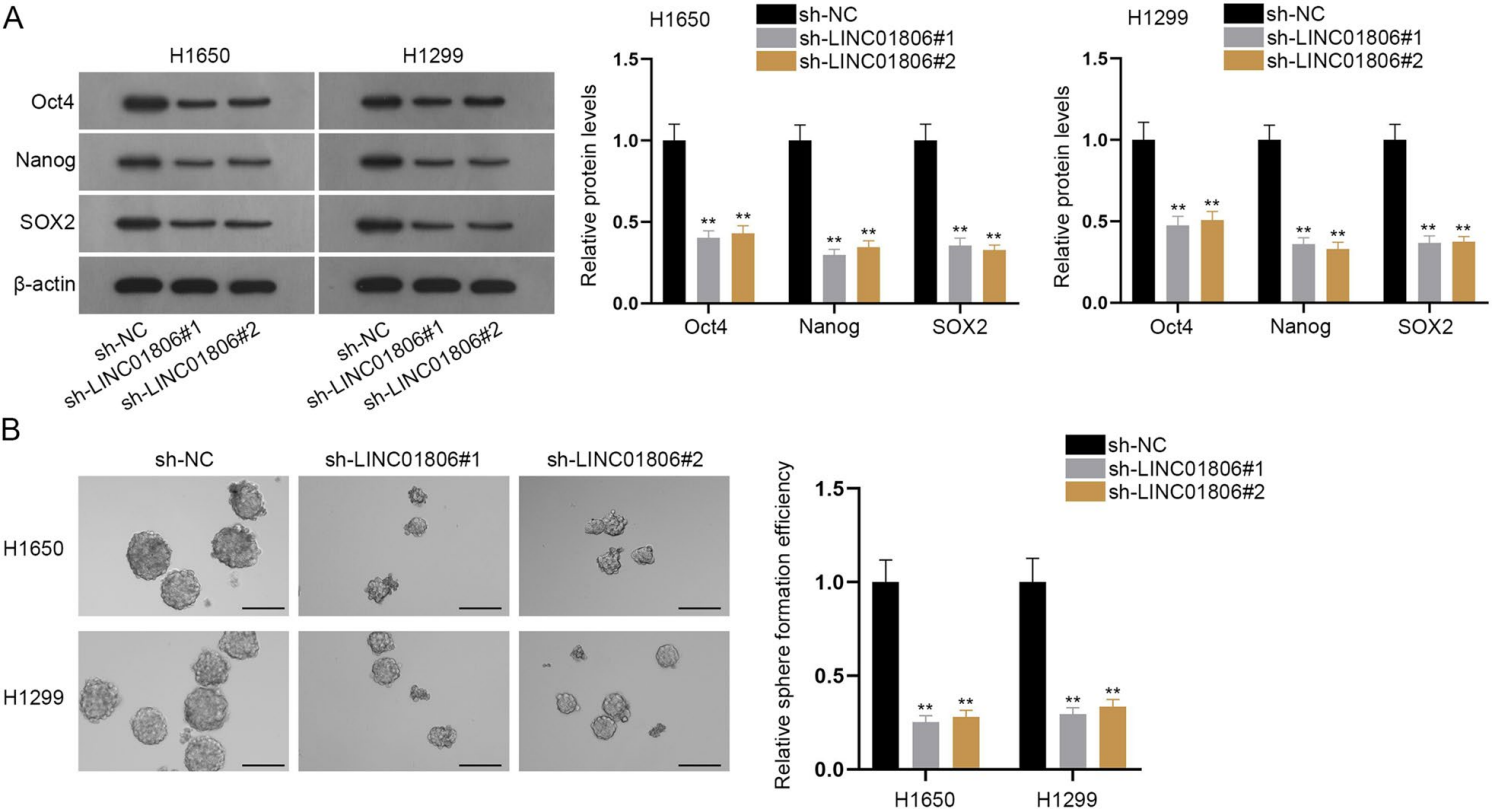
LINC01806 contributes to NSCLC cell stemness. **A** Western blot was utilized to measure the protein levels of stemness-linked genes (Oct4, Nanog, and SOX2) in NSCLC cells with or without LINC01806 depletion. **B** Sphere formation assay (scale bar = 100 μm) was performed to examine the sphere-forming capacity of NSCLC cells in response to LINC01806 deficiency. **P < 0.01

Meanwhile, we tested whether LINC01806 was also involved in the regulation of NSCLC cell stemness. Oct4, Nanog and SOX2 have been determined to be key factors of cell stemness [15]. Western blot analysis revealed that LINC01806 down-regulation reduced the protein level of Oct4, Nanog and SOX2 (Fig. 2A). Likewise, according to the results of sphere formation assay, we discovered that less and smaller spheres could be formed by LINC01806-deficient NSCLC cells, which meant that LINC01806 depletion lowered the sphere formation efficiency of NSCLC cells (Fig. 2B), which further testified that inhibition of LINC01806 attenuated cell stemness in NSCLC. The gain-of-function assays were performed in BEAS-2B cells after the overexpression efficacy of pcDNA3.1/LINC01806 was confirmed to be high (Additional file 1: Fig. S1B). The following western blots indicated that LINC01806 augment promoted BEAS-2B cell stemness (Additional file 1: Fig. S1C). The data from sphere formation assay also demonstrated that BEAS-2B cell stemness was enhanced by LINC01806 up-regulation (Additional file 1: Fig. S1D). To conclude, LINC01806 could facilitate NSCLC and BEAS-2 cell stemness.

### LINC01806 knockdown inhibits tumor growth in vivo

**Fig. 3:**
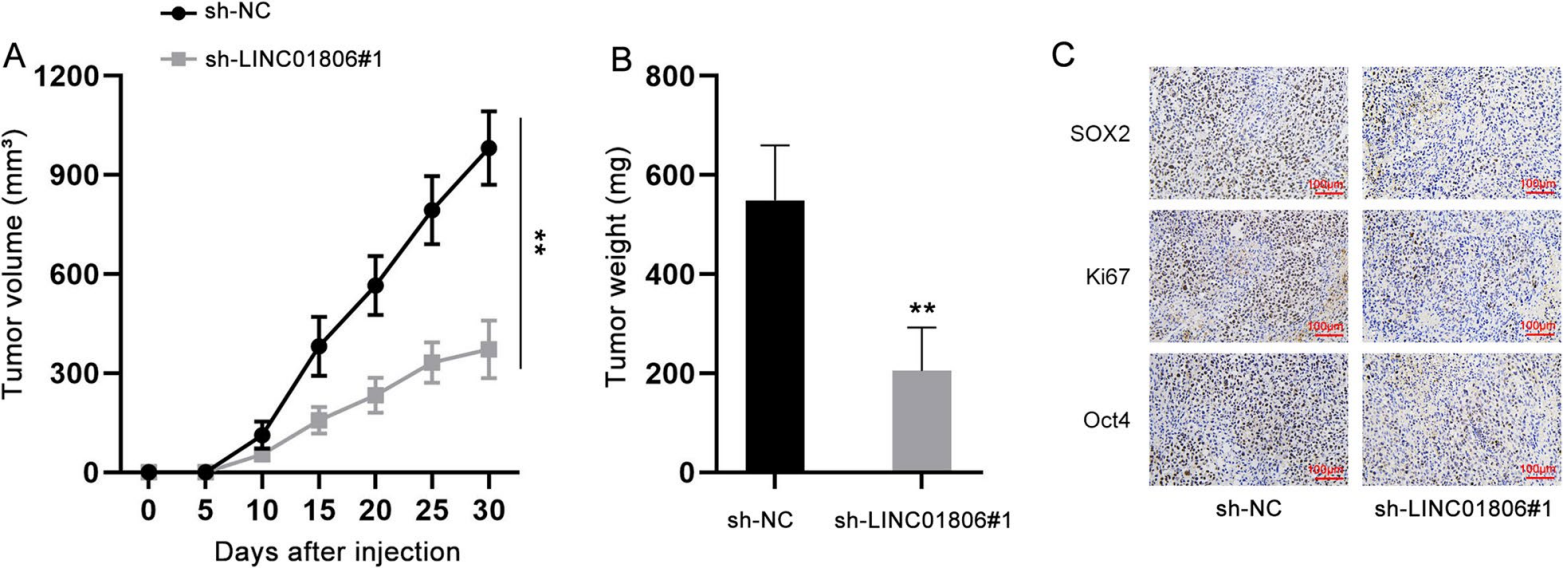
Silencing LINC01806 restrains NSCLC tumor growth in vivo. **A** Average volume of tumors in nude mice injected with control or LINC01806-silenced NSCLC cells was measured every 5 days. **B** The weight of tumors formed in indicated groups was calculated. C. IHC assay (scale bar = 100 μm) tested the expression of SOX2, Ki67 and Oct4 in tumors of different groups. **P < 0.01

Later, we injected NSCLC cells transfected with sh-NC or sh-LINC01806#1 into mice to assess the impact of LINC01806 on tumor growth. As demonstrated in Fig. 3A, the growth rate of tumor obviously slowed down when LINC01806 was silenced in NSCLC cells. Moreover, tumor weight was decreased on account of LINC01806 down-regulation (Fig. 3B). In addition, we tested SOX2, Ki67 and Oct4 expression in tumor tissues from different groups. Results indicated that the positivity of SOX2, Ki67 and Oct4 was reduced in tumor tissues with silenced LINC01806 (Fig. 3C & Additional file 1: Fig. S1E). Overall, LINC01806 works as a tumor promoter in NSCLC.

### STAT1 activates the transcription of LINC01806

**Fig. 4:**
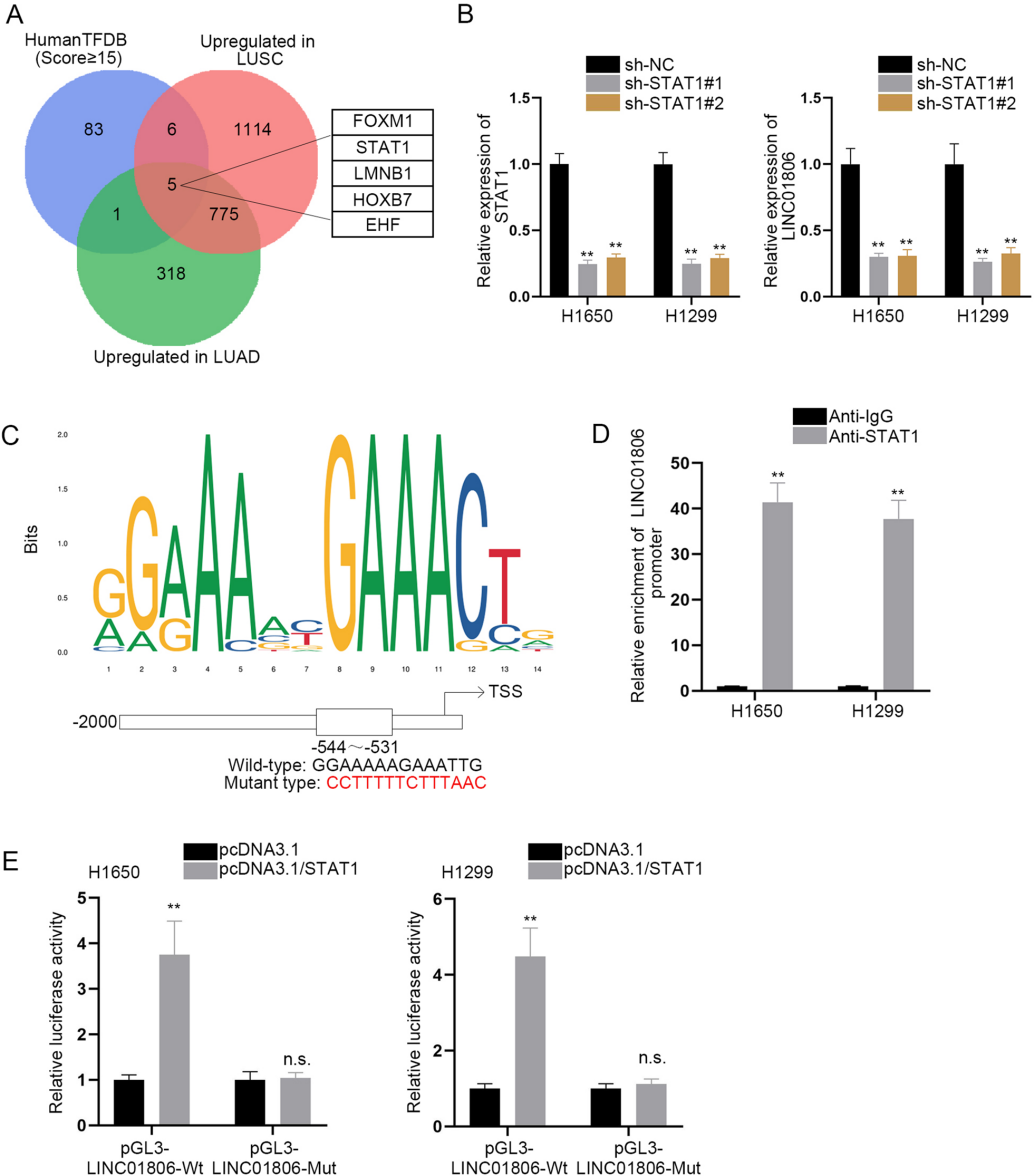
STAT1 activates the transcription of LINC01806. **A** Venn diagram depicted five TFs selected by GEPIA database and HumanTFDB. **B** RT-qPCR was performed to examine STAT1 and LINC01806 expression in NSCLC cells upon STAT1 knockout. **C** DNA motif of STAT1 along with one predicted binding site of STAT1 in LINC01806 promoter was predicted from JASPAR. **D** ChIP assay was utilized to test the binding affinity of STAT1 and LINC01806 promoter. **E** The combination of STAT1 and LINC01806 promoter was detected through luciferase reporter assay. **P < 0.01, *n.s.* no significance

Evidence has shown that dysregulation of lncRNAs in human malignancies can be caused by transcription factors (TFs) [16, 17]. Through the prediction of HumanTFDB (http://bioinfo.life.hust.edu.cn/HumanTFDB#!/), 95 TFs were found to possess potential binding with LINC01806 promoter with the score ≥ 15; further, only 5 TFs (FOXM1, STAT1, LMNB1, HOXB7, and EHF) among them were up-regulated in both LUSC and LUAD based on the data from GEPIA (Fig. 4A). To determine which candidate TFs might promote the transcription of LINC01806, RT-qPCR assays were applied to quantify the expression of LINC01806 before and after those TFs were overexpressed. The results indicated that only when STAT1 was up-regulated, LINC01806 expression was increased (Additional file 1: Fig. S1F). Thereby, the rest of the potential TFs were excluded. Subsequently, it was exhibited that STAT1 deficiency lessened the expression level of LINC01806 (Fig. 4B). Meanwhile, RT-qPCR and western blot analyses suggested that LINC01806 depletion made no influence on the mRNA and protein levels of STAT1 (Additional file 1: Fig. S1G). The putative binding site between STAT1 and LINC01806 promoter was projected on JASPAR website (http://jaspar.genereg.net/) (Fig. 4C). ChIP assay also displayed the high abundance of STAT1 in LINC01806 promoter region (Fig. 4D). Furthermore, the data from luciferase reporter assay reflected that STAT1 augment obviously elevated the luciferase activity of LINC01806 promoter-Wt, while having little impact on LINC01806 promoter-Mut activity (Fig. 4E), which signified STAT1 could bind to LINC01806 promoter and facilitate LINC01806 transcription. Taken together, STAT1 activates the transcription of LINC01806 in NSCLC cells.

### MiR-4428 is a downstream molecule of LINC01806 in NSCLC cells

**Fig. 5:**
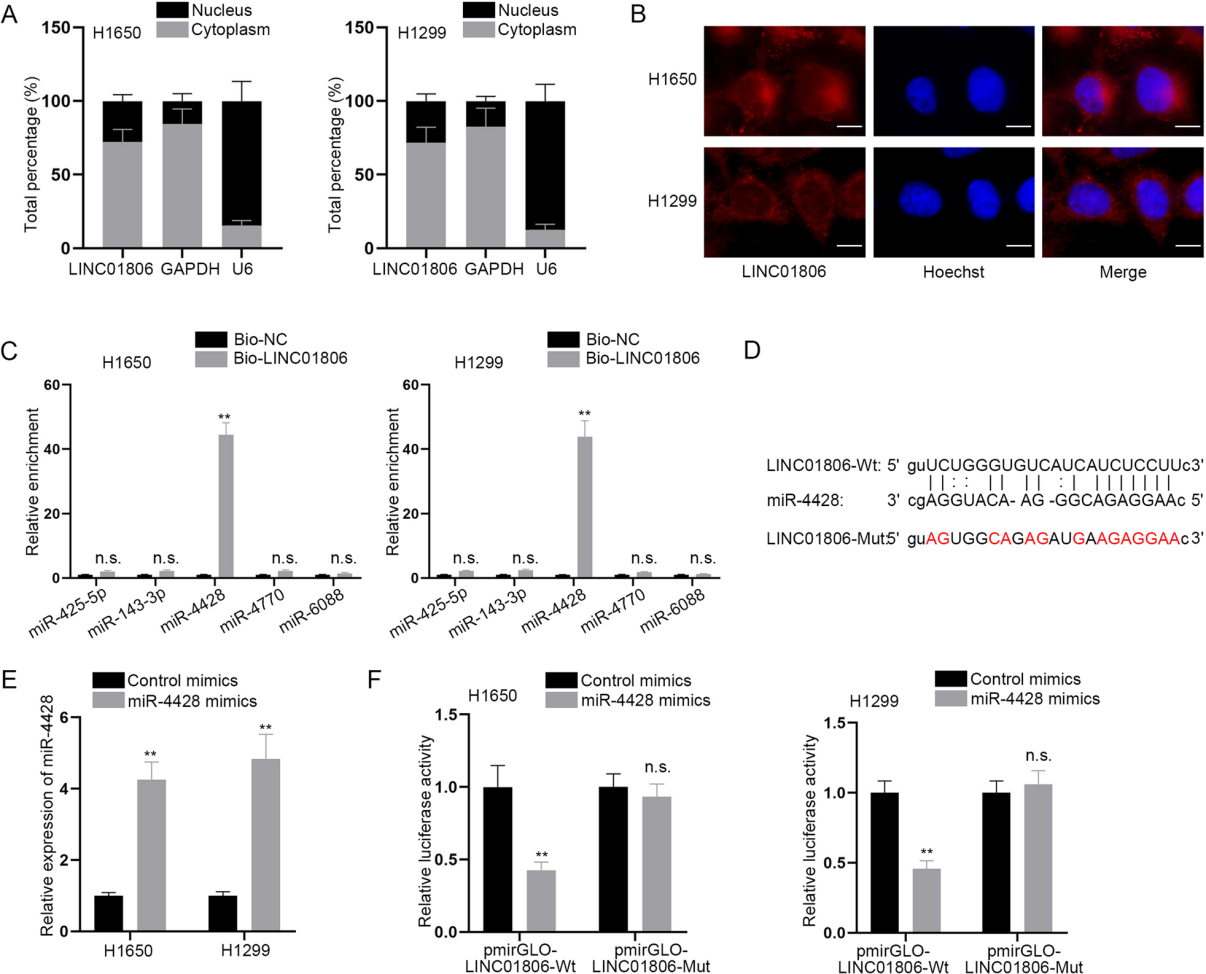
MiR-4428 is a downstream factor of LINC01806. **A**, **B**. Subcellular fractionation and FISH assays (scale bar = 10 μm) were carried on for the localization of LINC01806 in NSCLC cells. **C** The binding affinity of LINC01806 and candidate miRNAs was evaluated via RNA pull down assay. **D** StarBase projected the alignment between LINC01806 and miR-4428, and the mutated binding sites on LINC01806 were also shown. **E** The overexpression efficacy of miR-4428 mimics was tested via RT-qPCR. **F** The luciferase activity of LINC01806-Wt and LINC01806-Mut in H1650 and H1299 cells after transfection with miR-4428 mimics was detected through luciferase reporter assay. **P < 0.01, *n.s.* no significance

For deeply investigating the regulatory mechanism of LINC01806 in NSCLC cells, subcellular fractionation assay together with FISH assay was performed. The outcomes revealed LINC01806 was mostly amassed in NSCLC cell cytoplasm (Fig. 5A, B). Therefore, we presumed that LINC01806 might exert its roles in NSCLC cells by serving as a miRNA sponge. Using starBase (http://starbase.sysu.edu.cn/), we projected 5 miRNAs with the condition of CLIP Data ≥ 3. Then, RNA pull down assay verified that simply miR-4428 was abundantly precipitated in Bio-LINC01806 whereas other miRNAs were not (Fig. 5C). The potential miR-4428 binding sites within LINC01806 were predicted by starBase and presented in Fig. 5D. The expression level of miR-4428 in NSCLC cells was enhanced in response to the successful transfection of miR-4428 mimics (Fig. 5E). The findings of luciferase reporter assay represented that miR-4428 augment weakened the luciferase activity of LINC01806-Wt while failing to influence LINC01806-Mut activity (Fig. 5F), indicating the strong binding between miR-4428 and LINC01806. Additionally, the expression of miR-4428 after LINC01806 knockdown was tested by RT-qPCR, which was relatively stable (Additional file 2: Fig. S2A). In summary, LINC01806 functions as a sponge of miR-4428 in NSCLC cells.

### LINC01806 binds with miR-4428 to boost NOTCH2 expression and activate Notch signaling pathway

**Fig. 6:**
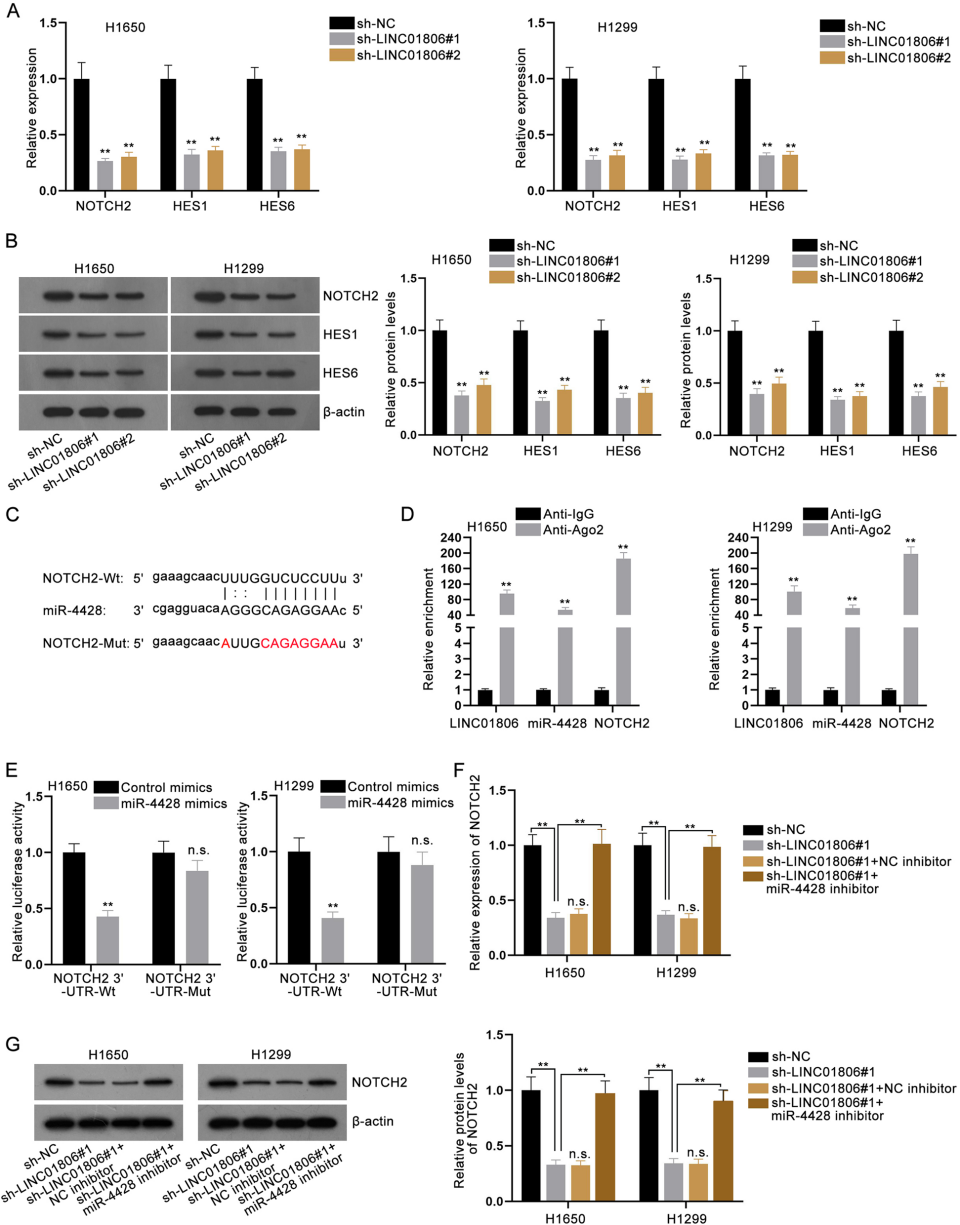
LINC01806 sponges miR-4428 to enhance NOTCH2 expression and activate Notch signaling pathway. **A**, **B** The levels of several factors related to Notch signaling pathway were examined via RT-qPCR and western blot in NSCLC cells after LINC01806 was silenced or not. **C** The binding sequence of miR-4428 and NOTCH2 projected by starBase, as well as the mutant NOTCH2 3’-UTR sequence, was exhibited. **D** RIP assay disclosed the levels of LINC01806, miR-4428 and NOTCH2 in Ago2-precipiated complex relative to that in control groups. **E** The luciferase activity changes of NOTCH2 3’-UTR-Wt and NOTCH2 3’-UTR-Mut were assessed via luciferase reporter assay in NSCLC cells with miR-4428 augment. **F**, **G** The expression of NOTCH2 was examined via RT-qPCR and western blot in NSCLC cells transfected with sh-NC, sh-LINC01806#1, sh-LINC01806#1 + NC inhibitor or sh-LINC01806#1 + miR-4428 inhibitor. **P < 0.01, *n.s.* no significance

Bioinformatics tools were utilized to project downstream targets of miR-4428. Interestingly, we discovered that NOTCH2, a main factor involved in Notch signaling pathway, was targeted by miR-4428. As reported, Notch signaling pathway is linked to the tumorigenesis, mutagenesis, as well as immune tolerance in NSCLC [18]. Hence, we chose NOTCH2 as our research object. To identify whether the oncogenic role of LINC01806 in NSCLC was concerned with Notch signaling pathway, we assessed its impact on several factors related to this pathway. As expected, we discovered the mRNA and protein levels of three crucial factors (NOTCH2, HES1 and HES6) associated with Notch signaling pathway were all lessened owing to LINC01806 knockdown (Fig. 6A, B). Moreover, miR-4428 overexpression also diminished the expression of those crucial factors (Additional file 2: Fig. S2B, C). Then we conjectured that LINC01806 might modulate NOTCH2 to stimulate Notch signaling pathway through competitively binding to miR-4428. The predicted alignment between miR-4428 and NOTCH2 was exhibited in Fig. 6C. RIP assay data disclosed the strong enrichment of LINC01806, miR-4428 and NOTCH2 in the precipitates captured by Ago2 antibody (Fig. 6D), which proved that all the three molecules could be enriched in RNA-induced silencing complex (RISC). In the meantime, the outcomes of luciferase reporter assay attested the binding affinity of miR-4428 and NOTCH2 3’-UTR at the putative sites (Fig. 6E). Furthermore, we noticed that the depressed levels of NOTCH2 on account of LINC01806 depletion were fully recovered by miR-4428 inhibition (Fig. 6F-G). The abovementioned results support that LINC01806 sequesters miR-4428 to enhance NOTCH2 expression, thus activating Notch signaling pathway.

### MiR-4428 inhibition or NOTCH2 up-regulation abrogates LINC01806 knockdown-induced repression on NSCLC cell proliferation, migration, invasion, and stemness

**Fig. 7:**
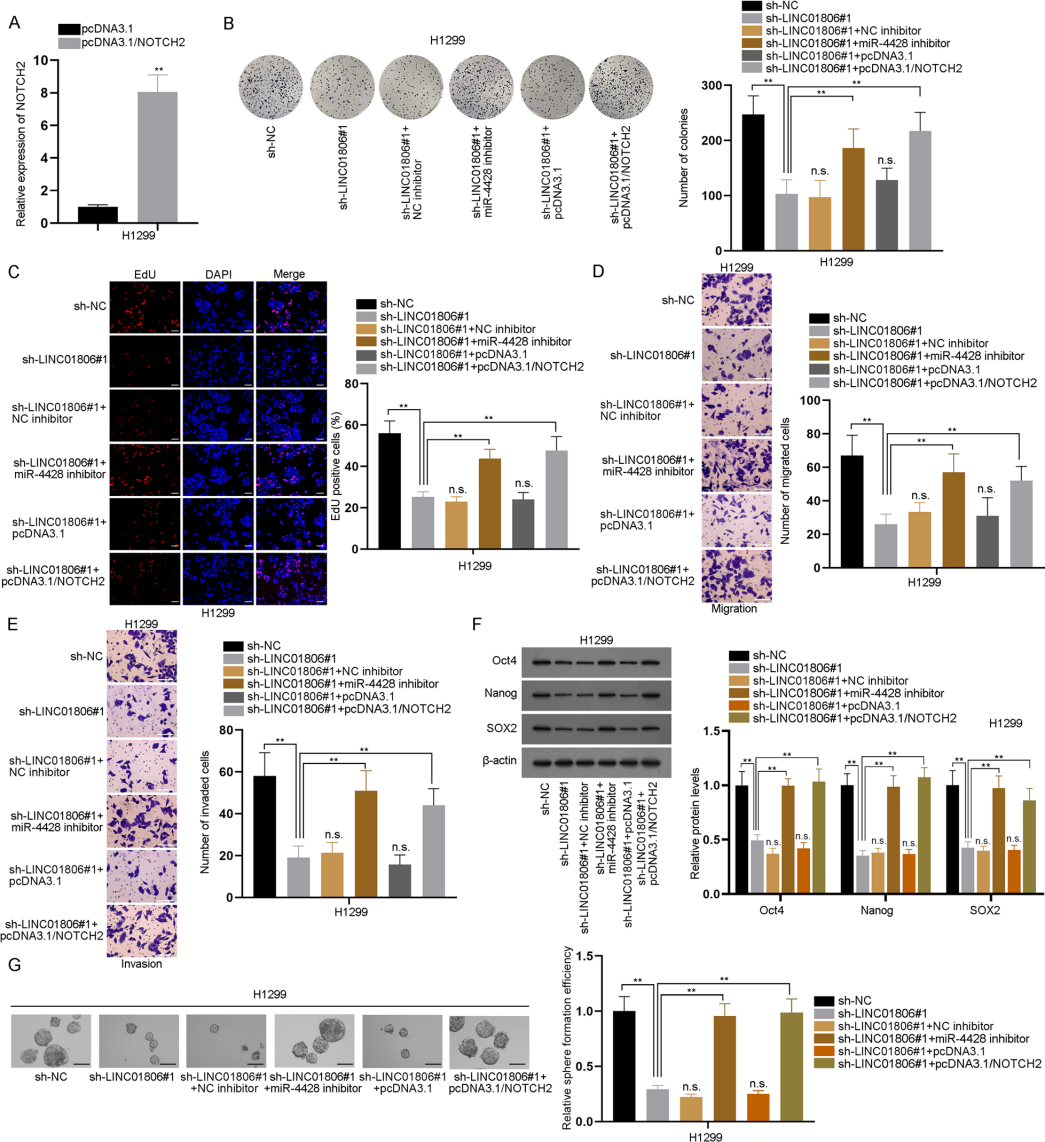
MiR-4428 down-regulation or NOTCH2 up-regulation abrogates LINC01806-knockdown-induced repression on NSCLC cell proliferation, migration, invasion and stemness. **A** RT-qPCR was performed to examine the efficacy of NOTCH2 overexpression. **B**, **C** The proliferative capacity of NSCLC cells was monitored in colony formation and EdU assays (scale bar = 100 μm) under various conditions (sh-NC, sh-LINC01806#1, sh-LINC01806#1 + NC inhibitor, sh-LINC01806#1 + miR-4428 inhibitor, sh-LINC01806#1 + pcDNA3.1, or sh-LINC01806#1 + pcDNA3.1/NOTCH2). **D**, **E** By transwell assay (scale bar = 100 μm), cell migration and invasion under different conditions were tested. **F** The expression of Oct4, Nanog and SOX2 was detected by western blot in H1299 cells after indicated transfections. **G** Cell stemness under indicated transfections was assessed by sphere formation assay (scale bar = 100 μm). **P < 0.01, *n.s.* no significance

To further explore whether LINC01806 affected NSCLC cell malignant behaviors via miR-4428/NOTCH2, a series of assays were done. At first, we augmented NOTCH2 expression (Fig. 7A). Seen from the data of colony formation and EdU assays, LINC01806 down-regulation restrained the proliferative capacity of NSCLC cells, while miR-4428 inhibition or NOTCH2 overexpression could fully offset its impact (Fig. 7B, C). In addition, transwell assay results also proved that inhibition of miR-4428 or up-regulation of NOTCH2 countervailed the suppression on cell migration and invasion because of LINC01806 depletion (Fig. 7D, E). Moreover, western blots manifested that the diminished protein levels of Oct4, Nanog, and SOX2 due to LINC01806 inhibition were all recovered by miR-4428 inhibition or NOTCH2 overexpression (Fig. 7F). Likewise, we observed that the attenuated sphere-forming ability of NSCLC cells caused by LINC01806 depletion was enhanced upon miR-4428 down-regulation or NOTCH2 overexpression (Fig. 7G). Collectively, LINC01806 facilitates the malignant phenotypes of NSCLC cells via targeting miR-4428/NOTCH2.

### LINC01806 mediates tumor growth in NSCLC via miR-4428/NOTCH2 pathway

**Fig. 8:**
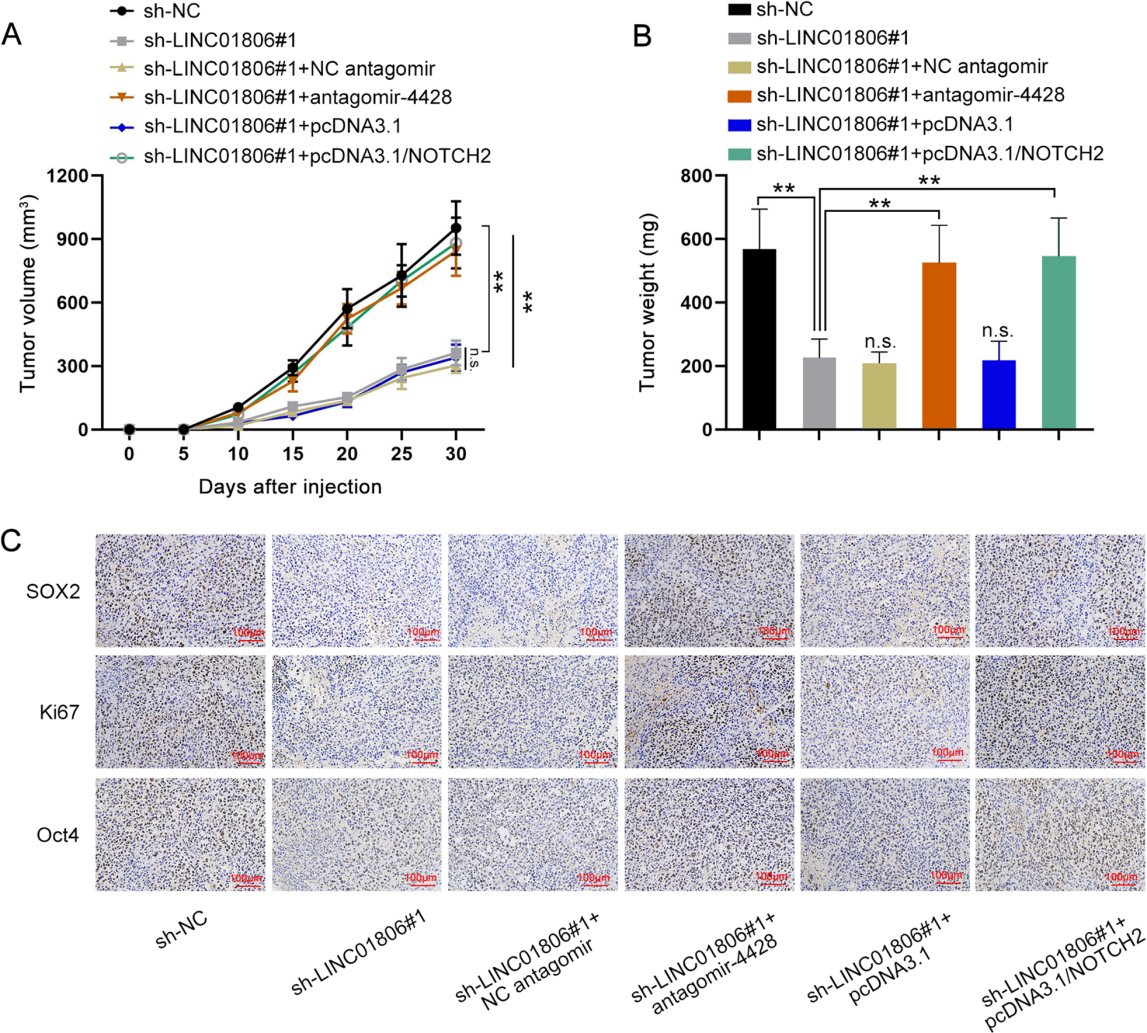
LINC01806 promotes tumor growth through targeting miR-4428/NOTCH2 axis. **A** Tumor volume in indicated groups was observed every five days. **B** Tumor weight in different groups was weighed. **C** IHC assay (scale bar = 100 μm) determined the positivity of SOX2, Ki67 and Oct4 in tumors from indicated groups. **P < 0.01, *n.s.* no significance

To further corroborate the interaction of LINC01806/miR-4428/NOTCH2 axis in NSCLC tumor growth, in vivo experiments were designed and performed. Mice were injected with NSCLC cells transfected with indicated plasmids. Xenograft tumor volume was observed every 5 days. Tumors were weighed after mice were sacrificed on the 30th day. As expected, the growth rate of tumors from LINC01806-silenced NSCLC cells slowed down compared to that in sh-NC group, whereas the reduction in tumor growth rate was restored when NSCLC cells were co-transfected with antagomir-4428 or pcDNA3.1/NOTCH2 (Fig. 8A). Consistently, the lessened weight of tumors originated from NSCLC cells with LINC01806 deficiency was recovered with further miR-4428 inhibition or NOTCH2 overexpression (Fig. 8B). Moreover, the reduced staining of SOX2, Ki67 and Oct4 in LINC01806-depleted tumors was restored in tumors with further inhibition of miR-4428 or up-regulation of NOTCH2 (Fig. 8C & Additional file 2: Fig. S2D). Furthermore, the expression of LINC01806 in obtained NSCLC tumors was also determined by ISH assay and we found that restricted LINC01806 expression induced by LINC01806 knockdown was not recovered by down-regulating miR-4428 or up-regulating NOTCH2 (Additional file 2: Fig. S2E). In conclusion, LINC01806 facilitates tumorigenesis in NSCLC via miR-4428/NOTCH2 pathway.

## Discussion

NSCLC has been characterized as a malignant epithelial lung cancer and also a primary cause of cancer-linked death around the world [1]. The stem cell properties of cancer cells are always closely linked to multiple processes, such as cancer recurrence, cell proliferation as well as drug resistance [19]. Namely, studying on cell stemness may be helpful for cancer treatment.

In recent years, more and more lncRNAs have been revealed to be aberrantly expressed and influence multiple malignant phenotypes in cancer cells. Our study first investigated LINC01806 and discovered that LINC01806 expression was increased in NSCLC cells. Additionally, LINC01806 inhibition restricted cell proliferative, migratory, invasive abilities along with stemness process in NSCLC. Moreover, depletion of LINC01806 retarded tumor growth in vivo.

As for the investigation of the upstream of LINC01806 in NSCLC, we surprisingly found that STAT1 served as a transcriptional activator of LINC01806, and it induced LINC01806 augment in NSCLC cells.

In the competing endogenous RNA (ceRNA) mechanism, lncRNAs regulate gene expression through sponging miRNAs. In present work, miR-4428 was recognized as the potential downstream of LINC01806 in NSCLC cells. MiR-4428 has been reported in advanced breast cancer [20]. However, it still remains elusive in most cancers. In this paper, we deeply delved into the interaction between LINC01806 and miR-4428. RNA pull down, RIP as well as luciferase reporter assays verified the binding of LINC01806 and miR-4428, certifying that miR-4428 was sponged by LINC01806 in NSCLC cells.

Notch signaling pathway has been uncovered to be aberrantly actuated in various cancers to affect multiple tumor processes, such as cell proliferation and metastasis [21, 22]. Also, the correlation between Notch signaling pathway and NSCLC development has been widely investigated. All results validated that the members of Notch signaling pathway may serve as potential biomarkers for NSCLC [23, 24]. As a crucial factor in Notch signaling pathway, NOTCH2 is indispensable in activating Notch signaling pathway. This study revealed that NOTCH2 was a target gene of miR-4428. Subsequently, experimental results substantiated that LINC01806 sponged miR-4428 to elevate the expression of NOTCH2, which finally motivated Notch signaling pathway in NSCLC. Moreover, the final in vitro and in vivo assays testified that LINC01806 facilitated NSCLC progression via targeting miR-4428/NOTCH2 pathway.

## Conclusion

LINC01806 was identified to be an oncogene in NSCLC since its knockdown hindered oncogenic phenotypes in vitro and blocked tumor growth in vivo. From the perspective of mechanism, LINC01806 was activated by STAT1; and it functioned as a sponge for miR-4428 to enhance NOTCH2 expression, consequently activating Notch signaling pathway. All these findings reflected that targeting LINC01806 might be a potential therapeutic approach for NSCLC.

## Supplementary Information

**Additional file 1: Figure S1**. A. Cell cycle was detected by flow cytometry analysis upon LINC01806 knockdown. B. Overexpression efficacy of pcDNA3.1-LINC01806 in BEAS-2B cells was assessed by RT-qPCR. C. Western blot assay measured the level of stemness-associated proteins in BEAS-2B cells. D. Sphere formation assay evaluated sphere formation ability of BEAS-2B cells. E. Quantitative results of Figure 3C were displayed. F. RT-qPCR was done to quantify the expression of LINC01806 after NSCLC cells were successfully transfected with the indicated plasmids: pcDNA3.1-FOXM1, pcDNA3.1-STAT1, pcDNA3.1-LMNB1, pcDNA3.1-HOXB7, and pcDNA3.1-EHF. G. STAT1 expression was examined by RT-qPCR and western blot when LINC01806 was knocked down. **P < 0.01.

**Additional file 2: Figure S2**. A. RT-qPCR assay was done to quantify miR-4428 expression after LINC01806 knockdown. B-C. The expression of NOTCH2, HES1, and HES6 was tested by RT-qPCR and western blot upon miR-4428 overexpression. D. Quantitative results of Figure 8C were presented. E. ISH assay was done to show the expression of LINC01806 in xenograft tumor tissues (scale bar = 100μm). **P < 0.01, n.s. no significance.

**Additional file 3: Table S1**. Sequences of shRNAs applied in this study were provided.

**Additional file 4: Table S2**. Sequences of primers involved in RT-qPCR were listed.

## Data Availability

Research data have all been presented within the manuscript and the additional files.
