# Supplementary figures and images for: LINC01806 mediated by STAT1 promotes cell proliferation, migration, invasion, and stemness in non-small cell lung cancer through Notch signaling by miR-4428/NOTCH2 axis
Source: Cancer Cell Int. 2022 May 22;22:198. doi: 10.1186/s12935-022-02560-8 (PMC9125941; doi:10.1186/s12935-022-02560-8)

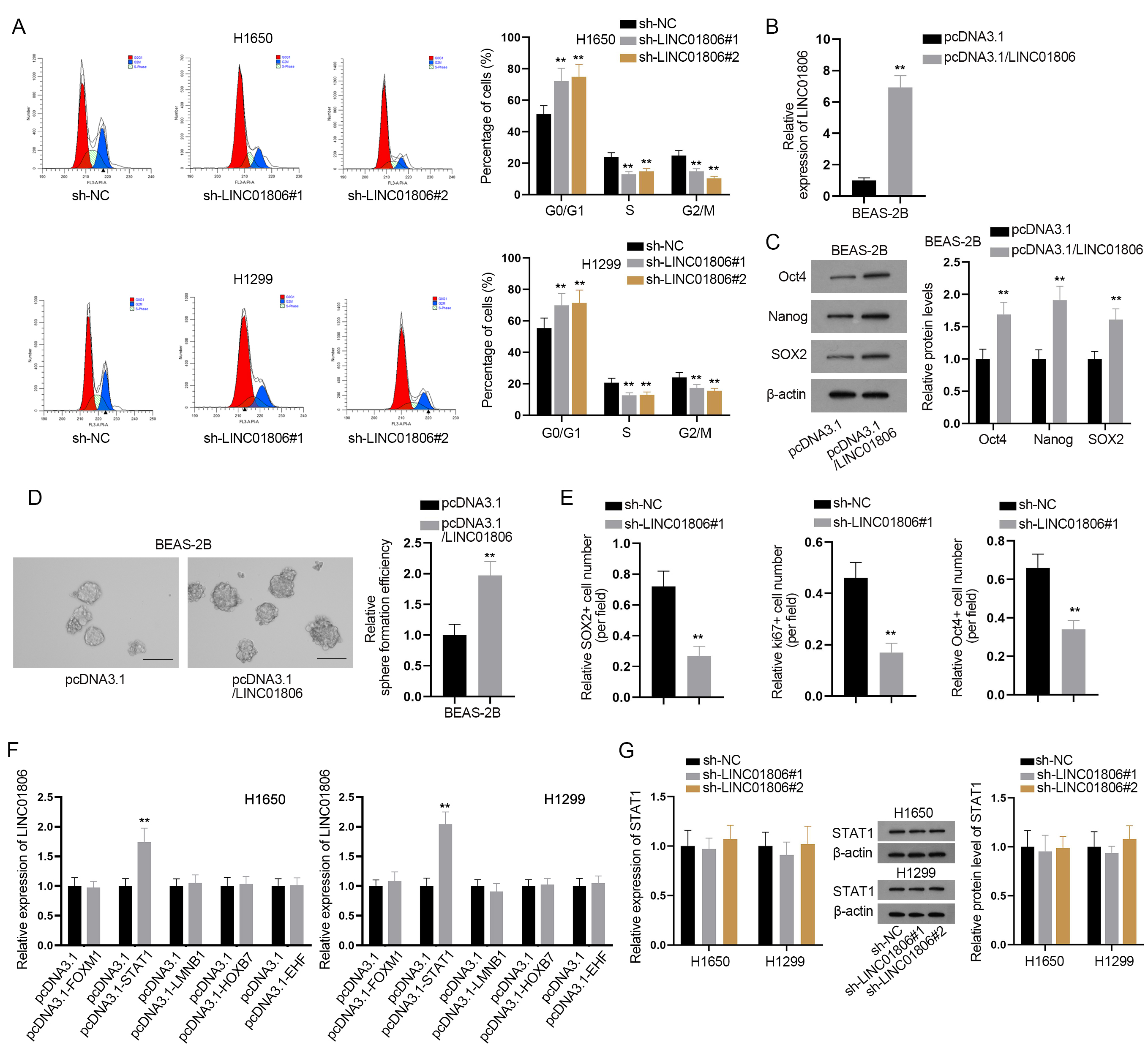

Supplement: Supplementary file 1 — Additional file 1: Figure S1. A. Cell cycle was detected by flow cytometry analysis upon LINC01806 knockdown. B. Overexpression efficacy of pcDNA3.1-LINC01806 in BEAS-2B cells was assessed by RT-qPCR. C. Western blot assay measured the level of stemness-associated proteins in BEAS-2B cells. D. Sphere formation assay evaluated sphere formation ability of BEAS-2B cells. E. Quantitative results of Figure 3C were displayed. F. RT-qPCR was done to quantify the expression of LINC01806 after NSCLC cells were successfully transfected with the indicated plasmids: pcDNA3.1-FOXM1, pcDNA3.1-STAT1, pcDNA3.1-LMNB1, pcDNA3.1-HOXB7, and pcDNA3.1-EHF. G. STAT1 expression was examined by RT-qPCR and western blot when LINC01806 was knocked down. **P < 0.01. [file 12935_2022_2560_MOESM1_ESM.jpg]

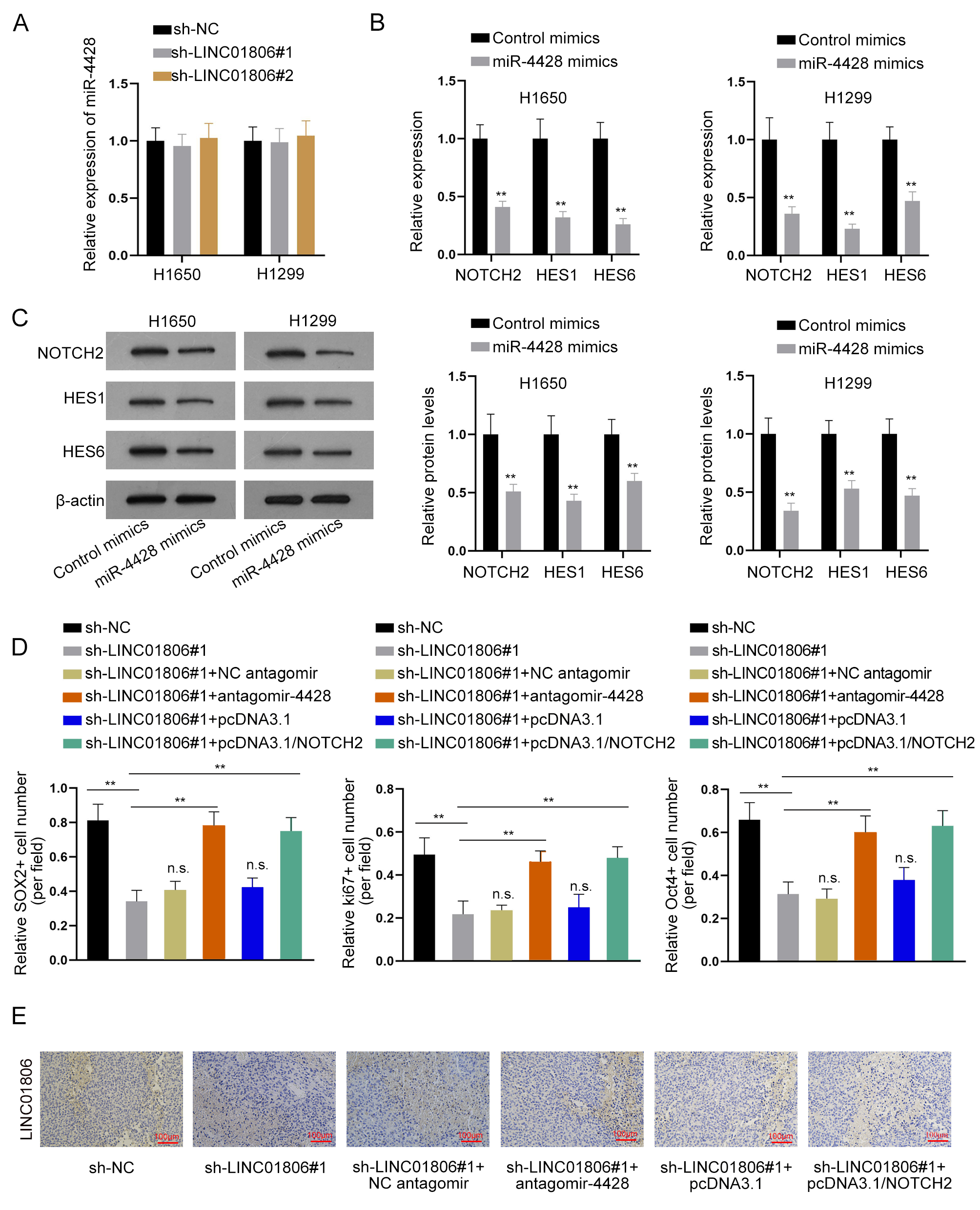

Supplement: Supplementary file 2 — Additional file 2: Figure S2. A. RT-qPCR assay was done to quantify miR-4428 expression after LINC01806 knockdown. B-C. The expression of NOTCH2, HES1, and HES6 was tested by RT-qPCR and western blot upon miR-4428 overexpression. D. Quantitative results of Figure 8C were presented. E. ISH assay was done to show the expression of LINC01806 in xenograft tumor tissues (scale bar = 100μm). **P < 0.01, n.s. no significance. [file 12935_2022_2560_MOESM2_ESM.tif]
