# Supplementary material for: LINC01806 mediated by STAT1 promotes cell proliferation, migration, invasion, and stemness in non-small cell lung cancer through Notch signaling by miR-4428/NOTCH2 axis
Source: Cancer Cell Int. 2022 May 22;22:198. doi: 10.1186/s12935-022-02560-8 (PMC9125941; doi:10.1186/s12935-022-02560-8)
Supplement: Supplementary file 3 — Additional file 3: Table S1. Sequences of shRNAs applied in this study were provided. [file 12935_2022_2560_MOESM3_ESM.pdf]

**Plasmids**	**Sequences**
sh-NC for sh-LINC0180[illegible]	CCGGTATTCTTTTCTGGCTACCTATCTCGAGATAGGTAGCCAGAAAAGAATATTTTTG
sh-LINC01806#1	CACCGTAATTTCTCCATTTTCTGGCTCTCGAGAGCCAGAAAATGGAGAAATTA
sh-LINC01806#2	CACCACGACATTCCTGTCAATGGGACTCGAGTCCCATTGACAGGAATGTCG
sh-LINC01806#3	CACCGTAGAACTTGTAAATATCTGTACTCGAGTACAGATATTTACAAGTTCTA
sh-NC for sh-STAT1	CCGGTGTTCTTATTGCTATCTTCGTCTCGAGACGAAGATAGCAATAAGAACATTTTTG
sh-STAT1#1	CACCGTGCTTTTCCTTATGTTATGCTCTCGAGAGCATAACATAAGGAAAAGCA
sh-STAT1#2	CACCGTTGATTTCATGCTCTATACACCTCGAGGTGTATAGAGCATGAAATCAA
sh-STAT1#3	CACCACATCTTCTTGAGTAACAGCTCTCGAGAGCTGTTACTCAAGAAGATG
